# The impact of preoperative interview and prospective nursing on perioperative psychological stress and postoperative complications in patients undergoing TACE intervention for hepatocellular carcinoma

**DOI:** 10.1097/MD.0000000000035929

**Published:** 2024-01-12

**Authors:** Leilei Gao, Wei Chen, Shuaixin Qin, Xi Yang

**Affiliations:** a Department of Radiology, Xingtai People’s Hospital, Interventional Catheter Room, Xingtai, Hebei, People’s Republic of China; b Department of Neurosurgery, Xingtai People’s Hospital, Xingtai, Hebei, People’s Republic of China.

**Keywords:** complications, liver cancer, preoperative visit, prospective nursing, psychological stress, transarterial chemoembolization

## Abstract

TACE has become one of the main methods for the treatment of liver cancer. The study aimed to investigate the effects of preoperative interview and prospective nursing in patients with hepatic carcinoma undergoing transcatheter chemoembolization (TACE). Eighty-six patients with hepatocellular carcinoma who underwent TACE intervention treatment at our hospital between 2020 and 2023 were selected and randomly assigned to 2 groups using computerized randomization. The control group (n = 43) received routine nursing care, while the study group (n = 43) received preoperative interviews in combination with prospective nursing during the procedure. The patients’ heart rate, mean arterial pressure, and blood pressure variations were recorded, along with their mood changes after intervention. The postoperative pain and satisfaction levels were compared between the 2 groups of patients, and the incidence of postoperative complications was observed. The heart rate, systolic blood pressure, and diastolic blood pressure of the 2 groups of patients were compared 1 day before the operation (*P* > .05). Compared to 1 day before the operation, there was no significant change for the study group at 10 minutes after entering the room. However, the control group showed an increase. Both groups showed an increase in heart rate, systolic blood pressure, and diastolic blood pressure after the operation, with the study group having lower values than the control group (*P* < .05). The levels of tension, fatigue, anxiety, energy, anger, depression, self-esteem, and POMS index were compared between the 2 groups before intervention (*P* > .05). After intervention, there were significant differences between the 2 groups(*P* < .05). Immediately after the operation, the NRS scores of the 2 groups of patients were compared (*P* > .05). Compared to the control group, the study group showed a decrease in NRS scores at 12, 24, and 48 hours after the operation (*P* < .05). The nursing satisfaction rate of the study group patients was 97.67% (42/43), which was higher than the nursing satisfaction rate of the control group of 76.74% (33/43) (*P* < .05). Compared to routine nursing, preoperative visits and prospective nursing interventions can effectively alleviate patients’ psychological stress reactions, relieve pain, reduce the incidence of complications, and improve patients’ satisfaction with nursing care.

## 1. Introduction

Primary liver cancer is a relatively prevalent malignant tumor in China, exhibiting a high incidence and mortality rate.^[1,2]^ With advancements in medical technology, TACE has emerged as a primary treatment method for liver cancer, especially for patients who are unable to undergo surgical resection or have metastatic liver cancer.^[3]^ However, psychological stress during the TACE procedure and postoperative complications remain important issues in perioperative management. This is because liver cancer itself can affect patients’ quality of life and cause a lot of physical discomfort. At the same time, patients with TACE experience impairments in their daily activities and quality of life due to various adverse symptoms. Those undergoing TACE often suffer from fatigue, depression, anxiety, reduced functional abilities, and sleep disorders.^[4,5]^ Routine nursing care focuses more on physiological care and technical operations, paying less attention to patients’ psychological needs and emotional support. Therefore, there is a need to optimize the nursing mode to improve the quality and effectiveness of nursing care.^[6]^

Preoperative visits serve as an early intervention strategy that equips patients with essential information and emotional support, enabling them to better manage the psychological stress preceding surgery.^[7]^ Prospective nursing involves the anticipation of potential complications before surgery, implementing targeted nursing interventions during and after the procedure to reduce complication rates, and enhance surgical outcomes.^[8–9]^ In light of these considerations, the primary objective of this study is to investigate the impact of combining preoperative visits with prospective nursing on mitigating psychological stress during the perioperative period of TACE intervention for liver cancer, as well as reducing postoperative complications.

## 2. Data and methods

### 2.1. General data

This study was approved by the Ethics Committee of Xingtai People Hospital, 86 liver cancer patients who underwent TACE intervention treatment at our hospital from February 2020 to February 2023were selected. The patients were randomly assigned to 2 groups using a computerized randomization method. The control group (n = 43) received standard nursing care, while the study group (n = 43) received preoperative visits in conjunction with proactive nursing during the surgery. General information about patients in both groups was collected, and no significant differences were found (*P* > .05). Please refer to Table 1 for details.

**Table 1:** Comparison of 2 groups of general data[*_ ± *±*s*, n/(%)].

Group	Gender		BMI (kg/m^2^)	TNM stage		Age (yr old)	Child liver function classification	
	Male	Female		III stage	IV stage		A class	B class
Control group (n = 43)	26 (60.47)	17 (39.53)	22.15 ± 2.16	28 (65.12)	15 (34.88)	48.66 ± 7.66	20 (46.51)	23 (53.49)
Study group (n = 43)	25 (58.14)	18 (41.86)	22.87 ± 2.08	30 (69.77)	13 (30.23)	48.58 ± 7.23	21 (48.84)	22 (51.16)
χ^2^/*t*	0.048		−1.574	0.212		0.050	0.047	
*P*	.826		.119	.645		.960	.829	

Inclusion criteria: the patient was diagnosed as primary liver cancer by clinical diagnosis and confirmed by imaging examination, patients with normal renal function can be treated with TACE intervention, the tumor was confined to the liver without distant metastasis, patients were not suitable for surgical resection, liver transplantation or radiofrequency ablation after preoperative evaluation, and patients were treated with TACE for the first time.

Exclusion criteria: the patient liver function was seriously damaged and could not bear the burden of TACE treatment, patients with tumor spread, through TACE interventional therapy cannot get effective treatment, patients with severe heart disease, lung disease or acute infection, and pregnant or lactating women.

### 2.2. Methods

The control group receives standard care: Preoperative anesthesia preparations are carried out, and patients are instructed to fast in accordance with anesthesia department requirements. Patients’ conditions are closely monitored during and after the procedure. Nutritional support and dietary guidance are provided during the recovery period.

The study group received a combination of preoperative visits and proactive care during the operation, which included the following components: Comprehensive Preoperative Assessment and Communication: Prior to the surgery, a thorough evaluation of the patient baseline health was conducted. Emphasis was placed on open and effective communication with patients while respecting their privacy and personal space. During these discussions, plain and easily understandable language, free from technical jargon, was used to ensure that patients fully understood the information provided. Patients were encouraged to voice their thoughts, questions, and concerns. Additionally, their level of understanding about liver cancer and the TACE procedure was assessed, and any misconceptions or gaps in knowledge were addressed. Customized explanations of the TACE procedure, its associated risks, and expected outcomes were provided, along with relevant information to address patient inquiries. Psychosocial Assessment and Counseling: Psychosocial assessments, including anxiety and stress assessments, were employed to measure patients’ levels of anxiety, fear, and psychological stress. Face-to-face conversations were held to observe patients’ facial expressions, language, and behavior, providing insight into their emotional state. This encompassed subtle changes such as tension or irritability, as well as emotional responses like crying or withdrawal. Positive psychological support was then extended to patients, with a dedicated professional psychologist or nurse available for consultations and communication at any time. Patients were encouraged to maintain a positive attitude and confidence, and their family and friends were guided to establish group contacts to offer additional emotional support. During psychological counseling, patients were given ample time and space to express their emotions and doubts, with a focus on using language and encouragement to convey positivity and enhance their emotional well-being. Patients were also encouraged to participate in relaxing activities like art therapy, music therapy, or meditation to strengthen their emotional and psychological equilibrium. Comprehensive Preoperative Assessment: A comprehensive assessment encompassing physiological parameters, laboratory tests, liver function, tumor size, and other pertinent information was conducted before the surgery. Prospective evaluation was employed to identify potential postoperative complications based on postoperative recovery indicators like consciousness level, pain levels, and the presence of any complications. Personalized nursing plans were then devised based on the assessment results, encompassing dietary management, wound care, urinary and bowel management, pain management, anti-infection treatment, and postoperative psychological care. Targeted and proactive care was implemented to alleviate postoperative pain symptoms, which can significantly impact patients’ recovery and quality of life. Common postoperative pain symptoms, such as pain at the incision site, were addressed. The standard pain assessment tool, the Numerical Rating Scale (NRS), was utilized to quantify the degree of pain, and the assessment results were recorded. Depending on the patient pain level and individual differences, appropriate pain management strategies were employed. This included the use of over-the-counter non-steroidal anti-inflammatory drugs or prescription analgesic medications like opioids or local anesthetics. Additionally, cold or hot compress treatments were offered to alleviate discomfort and reduce swelling at the surgical site. Relaxation techniques such as deep breathing, progressive muscle relaxation, or music therapy were employed to help reduce tension and anxiety, ultimately alleviating pain. Postoperative surgical procedures can affect gastrointestinal motility and digestive function, resulting in discomfort symptoms such as nausea, vomiting, abdominal distension, and constipation. Consequently, postoperative dietary management was introduced. This involved the creation of a suitable dietary plan that considered the type, timing, and quantity of meals. Nutrient-rich foods were provided to meet the patient energy and nutritional requirements, promoting wound healing and physical recovery. The use of anesthesia during surgery can impact the nervous system and muscles, potentially inhibiting urinary and bowel function. Therefore, urinary and bowel management protocols were implemented. The patient urination and defecation were monitored to ensure normal excretory function. To prevent postoperative infections, antimicrobial therapy was administered while adhering to hand hygiene and infection control principles. The patient temperature and signs of infection were closely monitored to detect and address potential infections promptly. Postoperative pain, discomfort, and maladjustment can have adverse psychological effects on patients. As a result, patient education and psychological support were provided. This included explaining postoperative precautions and promoting recovery measures, such as avoiding strenuous exercise, maintaining wound cleanliness and dryness, and adhering to medication schedules to prevent complications. Ongoing psychological communication with patients was maintained postoperatively. Their psychological distress and emotional expressions were actively listened to. Detailed information about postoperative recovery, including common physical changes, activity limitations, and rehabilitation plans, was provided to enhance their understanding and confidence in the recovery process.

### 2.3. Observation indicators

1. Changes in patients’ heart rate, systolic blood pressure, and diastolic blood pressure were documented at 3 time points: 1 day before surgery, 10 minutes after entering the operating room, and postoperatively.
2. Patients’ mood was assessed using the Profile of Mood States (POMS)^[10]^ scale before and after the intervention. This evaluation encompassed tension (score range 0–24), fatigue (score range 0–20), confusion (score range 0–20), vigor (score range 0–24), anger (score range 0–28), depression (score range 0–24), and self-esteem (score range 0–20). These 40 items were scored from 0 to 4 points each. The POMS index was calculated as the sum of scores for tension, depression, anger, confusion, and fatigue, minus the scores for vigor, self-esteem, and a correction term of 100. The score was inversely related to the patient mood state.
3. Postoperative pain in both patient groups was compared using the Numeric Rating Scale (NRS), which assesses pain on a scale of 0 to 10, with a score of 10 indicating severe pain.
4. Patient satisfaction was assessed using a self-designed satisfaction questionnaire. This questionnaire rated satisfaction on a scale of 0 to 4 for dissatisfaction, 5 to 8 for basic satisfaction, and 9 to 10 for high satisfaction. The patient satisfaction rate was calculated as the sum of patients who were basically satisfied and highly satisfied, divided by the total number of cases.
5. Observations were made regarding the occurrence of postoperative complications in patients.

### 2.4. Statistical processing

SPSS23.0 software was used for data processing. Chi-square test was performed on the patient count data such as gender ratio, Child liver function classification, TMN staging, and satisfaction survey, expressed as (n/%). The measurement data of patients such as heart rate, mean arterial pressure, systolic blood pressure and diastolic blood pressure, psychological score, pain score and other groups were tested by independent sample t test, and paired t test was used for intra-group comparison, expressed as (‾χ± s). In this study, α = 0.05 is the test level.

## 3. Results

### 3.1. Comparison of changes of heart rate, mean arterial pressure, systolic blood pressure and diastolic blood pressure at different time

**Figure 1.**
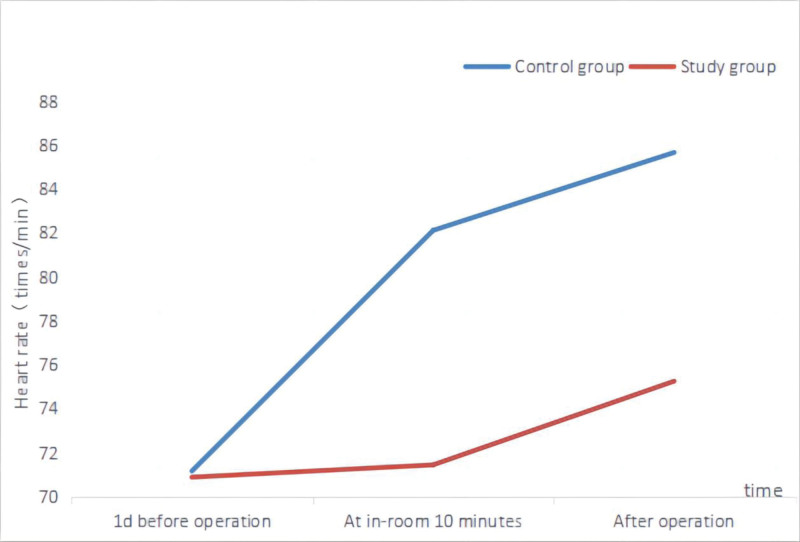
Two groups of heart rate changes at different times line chart.

**Figure 2.**
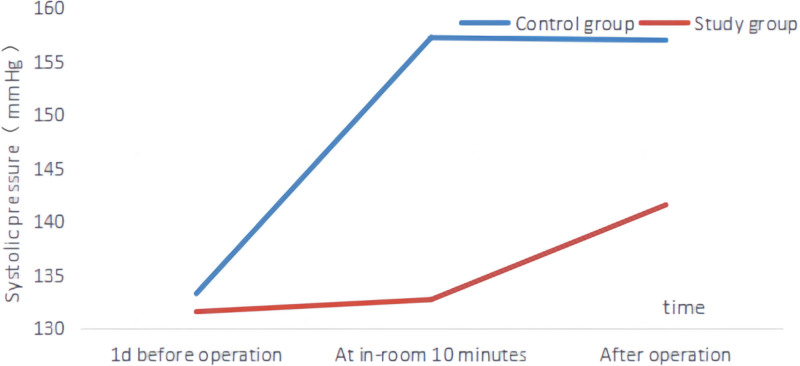
Two groups of different time systolic blood pressure changes line chart.

**Figure 3.**
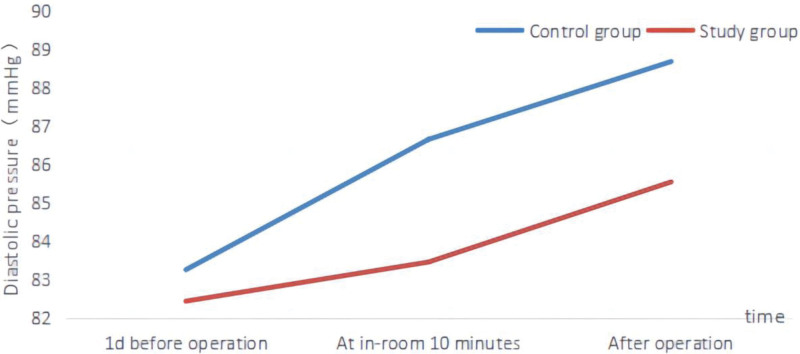
Two groups of different time diastolic blood pressure change line chart.

One day before the operation, there were no significant differences in the heart rate, systolic blood pressure, and diastolic blood pressure between the 2 groups (*P* > .05). When comparing the measurements taken in the operating room at 10 minutes after the operation to those 1 day before the operation, there were no significant changes in heart rate, systolic blood pressure, and diastolic blood pressure in the study group. However, the control group exhibited an increase in heart rate, systolic blood pressure, and diastolic blood pressure, while the study group values remained lower than those of the control group (*P* > .05). Following the operation, both groups experienced an increase in heart rate, systolic blood pressure, and diastolic blood pressure. Notably, the study group displayed lower values compared to the control group (*P* < .05). Please consult Table 2, Figures 1, 2, and 3 for detailed data.

**Table 2:** Comparison of changes of heart rate, mean arterial pressure, systolic blood pressure and diastolic blood pressure at different time (*_ ± *±*s*).

Group	Heart rate (times/min)			Systolic pressure (mm Hg)			Diastolic pressure (mm Hg)		
	1d before operation	At in-room 10 min	After operation	1d before operation	At in-room 10 min	After operation	1d before operation	At in-room 10 min	After operation
Control group (n = 43)	71.16 ± 8.12	82.11 ± 5.16*	85.66 ± 5.05*	133.25 ± 8.18	157.22 ± 5.49*	156.98 ± 5.48*	83.26 ± 5.98	86.66 ± 3.17*	88.69 ± 2.49*
Study group (n = 43)	70.88 ± 7.11	71.44 ± 6.33	75.25 ± 6.23*	131.55 ± 8.45	132.68 ± 4.27*	141.55 ± 6.98*	82.44 ± 6.46	83.46 ± 3.36	85.55 ± 2.15*
*t*	0.170	8.567	8.512	0.948	23.137	11.402	0.611	4.543	6.259
*P*	.865	<.001	<.001	.346	<.001	<.001	.543	<.001	<.001

* Compared with 1 d before operation, *P* < .05

### 3.2. Comparison of mood state scores between the 2 groups before and after intervention

Before the intervention, there were no significant differences in the levels of tension, fatigue, anxiety, energy, anger, depression, self-esteem, and POMS (Profile of Mood States) score between the 2 groups (*P* > .05). However, after the intervention, the study group exhibited a significant decrease in tension, fatigue, anxiety, anger, depression, and POMS score compared to the control group, along with an increase in energy and self-esteem scores (*P* < .05). Detailed information can be found in Table 3.

**Table 3:** Comparison of mood state scores between the 2 groups before and after intervention (*_ ± *±*s*, score).

Group	Tension		Anger		Fatigue		Depression	
	Before intervention	After intervention	Before intervention	After intervention	Before intervention	After intervention	Before intervention	After intervention
Control group (n = 43)	16.99 ± 3.06	14.22 ± 2.60*	20.11 ± 4.06	17.23 ± 3.04*	15.95 ± 3.05	12.56 ± 2.19*	18.99 ± 3.98	16.44 ± 2.95*
Study group (n = 43)	16.55 ± 3.12	12.44 ± 2.11*	19.89 ± 3.64	14.15 ± 3.16*	15.66 ± 3.16	10.36 ± 2.09*	18.48 ± 3.08	14.55 ± 2.19*
*t*	0.660	3.486	0.265	4.606	0.433	4.766	0.665	3.373
*P*	.511	.001	.792	<.001	.666	<.001	.508	.001
Group	Anxiety		Energy		Self-esteem		POMS index	
	Before intervention	After intervention	Before intervention	After intervention	Before intervention	After intervention	Before intervention	After intervention
Control group (n = 43)	14.66 ± 3.19	12.77 ± 2.46*	14.99 ± 2.61	16.77 ± 3.09*	12.05 ± 2.12	14.55 ± 2.77*	158.98 ± 12.08	141.22 ± 8.97*
Study group (n = 43)	14.33 ± 3.29	10.38 ± 2.09*	14.51 ± 2.56	19.56 ± 3.07*	12.22 ± 2.19	16.18 ± 3.31*	154.89 ± 11.29	125.69 ± 12.07*
*t*	0.472	4.855	0.861	−4.200	−0.366	−2.476	1.622	6.772
*P*	.638	<.001	.392	<.001	.715	.015	.109	<.001

POMS = Profile of Mood States.

* Compared with before intervention, *P* < .05.

### 3.3. Comparison of postoperative NRS scores between the 2 groups

**Figure 4.**
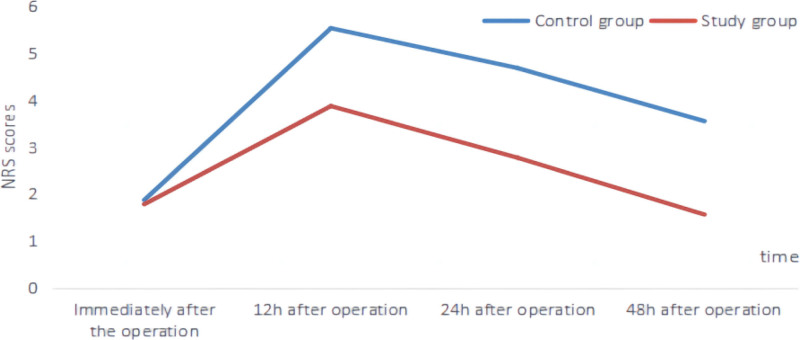
Line chart of NRS score changes at different time points in the 2 groups. NRS = Numerical Rating Scale.

Immediately after the operation, there was no significant difference in the NRS scores between the 2 patient groups (*P* > .05). However, when comparing NRS scores at 12, 24, and 48 hours after the operation, the study group showed significantly lower scores compared to the control group (*P* < .05). For detailed data, please refer to Table 4 and Figure 4.

**Table 4:** Comparison of postoperative NRS scores between the 2 groups (_ ± ±s).

Group	Immediately after the operation	12 h after operation	24 h after operation	48 h after operation
Control group (n = 43)	1.88 ± 0.78	5.54 ± 1.23	4.69 ± 1.18	3.56 ± 0.87
Study group (n = 43)	1.79 ± 0.69	3.88 ± 1.26	2.78 ± 1.16	1.57 ± 0.88
*t*	0.567	6.182	7.569	10.545
*P*	.572	<.001	<.001	<.001

### 3.4. Comparison of patient satisfaction between 2 groups

The nursing satisfaction rate among patients in the study group was 97.67% (42/43), significantly higher than the nursing satisfaction rate in the control group, which was 76.74% (33/43) (*P* < .05). For detailed statistics, please refer to Table 5.

**Table 5:** Comparison of patient satisfaction between 2 groups (n/%).

Group	Satisfied	Basically satisfied	Dissatisfied	Satisfied rate
Control group (n = 43)	21 (48.84)	12 (27.91)	10 (23.25)	33 (76.74)
Study group (n = 43)	32 (74.41)	10 (23.26)	1 (2.33)	42 (97.67)
*Z/*χ^2^	2.803			8.444
*P*	.005			.004

### 3.5. Comparison of postoperative complications between the 2 groups

In the study, 22 patients in the study group and 33 patients in the control group experienced gastrointestinal reactions after the operation. The incidence rate in the study group was significantly lower than that in the control group (*P* < .05). However, when comparing the occurrence of fever, dysuria, abdominal pain, intractable vomiting, and bleeding at the puncture site, there were no statistically significant differences between the 2 groups (*P* > .05). Please refer to Table 6 for detailed information.

**Table 6:** Comparison of postoperative complications between the 2 groups (n/%).

Group	Fever	Gastrointestinal reactions	Dysuria	Abdominal pain	Intractable vomiting	Bleeding at the puncture site
Control group (n = 43)	26 (60.47)	33 (76.74)	16 (37.21)	38 (88.37)	5 (11.63)	2 (4.65)
Study group (n = 43)	21 (48.84)	22 (51.16)	8 (18.60)	31 (72.09)	1 (2.33)	0 (0.00)
*Z/*χ^2^	1.173	6.103	3.699	3.592	2.867	2.048
*P*	.279	.013	.054	.058	.090	.152

## 4. Discussion

One of the main treatment methods for liver cancer is TACE, which involves injecting chemotherapy drugs and embolic agents into the hepatic artery to inhibit tumor growth and promote tumor necrosis.^[11,12]^ This procedure involves multiple stages, including preoperative preparation, intraoperative procedures, and postoperative recovery. These stages can lead to lifestyle changes and role transitions for patients, such as dietary restrictions, limited physical activity, and reduced social activities, which may cause some psychological stress for patients. In addition, uncertainty about the surgical outcome and life expectancy can also cause negative emotions such as anxiety and depression for patients.^[13,14]^ Preoperative nursing visit refers to the comprehensive assessment of patients’ personal conditions and psychological needs through establishing good communication and trust with patients. It aims to provide targeted surgical information and address patients’ concerns while providing appropriate emotional support.^[15,16]^ On the other hand, anticipatory nursing aims to predict possible complications and provide corresponding nursing measures during and after surgery to reduce the incidence of complications and improve surgical outcomes. Although some studies have focused on the effects of preoperative nursing visits or anticipatory nursing on the perioperative psychological status and postoperative complications, there are relatively few studies on the combined application of these 2 interventions. Therefore, it is necessary to systematically study and explore the effectiveness of the combined use of preoperative nursing visits and anticipatory nursing during the perioperative period of TACE for liver cancer.^[17,18]^ This study has certain limitations that should be acknowledged. Firstly, the sample size is relatively small, and increasing the sample size could enhance the accuracy and generalizability of the results. Secondly, the study may be constrained by the limited duration for assessing long-term impacts. It primarily focuses on data collected 1 day before surgery and 48 hours after surgery, with a short follow-up period. However, these do not affect the accuracy of the results in this article. In future research endeavors, we intend to address these limitations by expanding the sample size and extending the follow-up duration. This will enable a more comprehensive evaluation of patients’ psychological well-being and postoperative complications beyond their discharge from the hospital.

Although TACE surgery can have good therapeutic effects in inhibiting tumors, it can cause pain and trauma to patients, which can lead to fear and anxiety for some patients. In addition, patients who are undergoing the surgery for the first time may be unfamiliar and frightened by the operating room environment, the surgical process, and the physiological reactions after the surgery, which can easily cause emotional fluctuations and affect their blood dynamics.^[19,20]^ The results of this study showed that the heart rate, systolic blood pressure, and diastolic blood pressure of the group that received preoperative nursing visit and anticipatory nursing did not change significantly compared to 1 day before surgery, but were lower than the control group (*P* > .05). After surgery, the heart rate, systolic blood pressure, and diastolic blood pressure of both groups increased, but were lower in the study group than in the control group. This suggests that preoperative nursing visits combined with anticipatory nursing can stabilize patients’ emotions before and after surgery, thereby alleviating the hemodynamic fluctuations caused by patients’ fear and anxiety. This is because preoperative nursing visits combined with anticipatory nursing conduct a comprehensive evaluation of the patient understanding and the progression of their illness before surgery which helps patients to understand and prepare for the surgery, and can also raise relevant questions about the surgery.^[21,22]^ Meanwhile, nursing staff can explain the surgical process, risks, and expected outcomes to patients in detail based on the patients’ cognitive level. This increases patients’ understanding and knowledge of the disease and can improve their acceptance of the surgery. In addition, after becoming familiar with the procedure and expected results, it can also alleviate the fear and anxiety before and after the surgery.^[23–26]^

The results of this study showed that after the intervention, compared with the control group, the study group had reduced levels of tension, fatigue, confusion, anger, depression, and POMS index, while energy and self-esteem scores increased (*P* < .05). This suggests that preoperative nursing visits combined with anticipatory nursing can have a positive impact on patients’ psychological state. The reason for this is that the study group was able to assess patients’ emotional state and subtle changes more objectively by using standard psychological evaluation tools and observing non-verbal clues such as facial expressions, language and behavior. They were able to better evaluate patients’ anxiety, fear, and psychological stress levels as a baseline for intervention. Moreover, preoperative nursing visits combined with anticipatory nursing have provided patients with professional psychological counseling, assistance in establishing family group contacts, and encouragement to participate in relaxation activities such as art therapy, music therapy, or meditation to enhance emotional and psychological balance. These activities can help patients relax and reduce feelings of tension and anxiety.^[27–30]^

TACE intervention surgery is an invasive procedure, and the surgical incision can cause varying degrees of postoperative pain in patients. Additionally, the surgical process may stimulate the digestive system, thereby affecting postoperative digestive function. Furthermore, patients with chronic diseases or weak physical conditions may be more prone to increased psychological pressure and negative emotions. This study also showed that compared with the control group, the study group had lower NRS scores at different time periods after surgery, a lower incidence of gastrointestinal reactions than the control group (*P* < .05), and a higher level of nursing satisfaction. This suggests that preoperative nursing visits combined with anticipatory nursing have a good nursing effect on postoperative pain management and reducing the incidence of adverse reactions, leading to higher nursing satisfaction. This is because preoperative nursing visits combined with anticipatory nursing can use regular pain evaluation tools such as NRS to understand patients’ pain levels in a timely manner and take corresponding medication management measures. The correct selection and use of medication can alleviate pain, improve patients’ comfort and quality of life.^[31–33]^ In addition, relaxation techniques such as deep breathing, progressive muscle relaxation, and music therapy can reduce pain perception by improving patients’ psychological state and reducing tension and anxiety. These techniques can promote physical and psychological relaxation and reduce pain perception and reaction. By developing appropriate dietary plans and providing nutrient-rich foods, the incidence of gastrointestinal reactions can be reduced by meeting the energy and nutritional needs of patients.^[34,35]^

In summary, compared with routine care, the study group of patients who underwent preoperative nursing visits combined with anticipatory nursing had smaller changes in heart rate, mean arterial pressure, systolic pressure and diastolic pressure before and during surgery. In addition, the study group of patients had decreased mood indicators such as tension, fatigue, agitation, anger, and depression after surgery, along with improved scores in energy and self-esteem. Preoperative nursing visits combined with anticipatory nursing can effectively alleviate patients’ psychological stress reactions, relieve pain levels, reduce the incidence of complications and increase patients’ satisfaction with nursing care.

## Author contributions

**Conceptualization:** Leilei Gao, Wei Chen.

**Formal analysis:** Shuaixin Qin.

**Investigation:** Xi Yang.

**Supervision:** Wei Chen.

**Writing – original draft:** Leilei Gao.

**Writing – review & editing:** Leilei Gao, Wei Chen.
